# AT1R A1166C variants in patients with type 2 diabetes mellitus and diabetic nephropathy

**DOI:** 10.12860/jnp.2015.14

**Published:** 2015-07-01

**Authors:** Mahmoudreza Moradi, Zohreh Rahimi, Sonia Amiri, Ziba Rahimi, Mahmood Vessal, Hamid Nasri

**Affiliations:** ^1^Department of Urology and Regenerative Medicine, Kermanshah University of Medical Sciences, Kermanshah, Iran; ^2^Medical Biology Research Center, Kermanshah University of Medical Sciences, Kermanshah, Iran; ^3^Department of Biochemistry, Medical School, Kermanshah University of Medical Sciences, Kermanshah, Iran; ^4^Department of Biochemistry, Fars Science and Research Branch, Islamic Azad University, Fars, Iran; ^5^Department of Nephrology, Isfahan University of Medical Sciences, Isfahan, Iran

**Keywords:** AT1R A1166C polymorphism, Type 2 diabetes mellitus, Diabetic nephropathy, Macroalbuminuria

## Abstract

*Background:* There are inconsistent reports related to the role of angiotensin II type 1 receptor (AT1R) on the risk of type 2 diabetes mellitus (T2DM) and its renal complications.

*Objectives:* To identify the association between AT1R A1166C variants with the risk of T2DM and also with diabetic nephropathy (DN). Patients and Methods: In a case-control study, the AT1R A1166C polymorphism was detected in 135 T2DM patients with and without DN and in 98 healthy subjects from Western Iran. The genotypes of AT1R A1166C polymorphism were detected using polymerase chain reaction-restriction fragment length polymorphism (PCR-RFLP) method.

*Results:* The frequencies of AT1R A1166C genotypes and alleles were not significantly difference between patients with and without DN and controls. The frequencies of rare allele of 1166 C were 10%, 16.5%, 15.9% and 15.3% in micro-, macro- and normo-albuminuric patients and in healthy individuals, respectively ( *P* > 0.05). The systolic blood pressure and serum creatinine level in DN patients were significantly higher in carriers of AT1R CC compared to carriers of AT1R AA genotype. In the presence of uncontrolled hyperglycemia (HbA1c > 7.5%), there was a trend toward increased risk of macro-albuminuria in carriers of AC+CC genotype (OR=3.66, [95% CI: 0.81-16.58], *P* = 0.092).

*Conclusions:* Our study indicated the absence of an association between AT1R A1166C polymorphism with the risk of T2DM and DN. It seems in carriers of AT1R C allele systolic blood pressure and serum creatinine level to be higher compared to the A allele carriers.

**Implication for health policy/practice/research/medical education:**

The AT1R variants were not directly associated with the risk of T2DM or DN. However, the AT1R AC+CC genotype tended to increase the risk of macro-albuminuria in the presence of HbA1c >7.5%. Also, in carriers of AT1R 1166 C allele the systolic blood pressure and serum creatinine level were higher compared to the 1166 A allele carriers.

## 1. Background

Diabetes mellitus (DM) is the main cause of end-stage renal disease (ESRD) in both developed and developing countries (1). Both genetic and environmental factors are involved in the pathogenesis of type 2 diabetes mellitus (T2DM), the most common form of diabetes, and its micro- and macro-vascular complications (2). The renin angiotensin aldosterone system (RAAS) that could be activated by hyperglycemia plays a central role in the regulation of sodium metabolism, vascular tone, blood pressure, renal hemodynamics, and vascular modeling (3). The presence of hyperglycemia in diabetic patients increases tissue angiotensin II (Ang II) which induces oxidative stress, glomerular hyperfiltration, endothelial damage, thrombosis, inflammation and vascular remodeling (4). In patients with T2DM the inhibition of RAAS reduced the progression from normo- to micro- and micro- to macro-albuminuria and slowed the development of ESRD (1).

Ang II binds to 2 main types of receptors ofangiotensin II type 1 receptor (AT1R) and the angiotensin II type 2 receptor (AT2R). The vasoconstriction and the proliferative action of Ang II is performed through AT1R, while the AT2R inhibits cell proliferation, mediates apoptosis and works as a cardioprotective agent against AT1R (5). The cellular effects of Ang II in adult humans are mainly mediated by the AT1R (5).

Diabetic nephropathy (DN) starts with various renal functional changes including glomerular hyperfiltration and hyperperfusion, and is manifested with micro-albuminuria that subsequently can progress to macro-albuminuria (6). DN and ESRD diseases are major causes of mortality in DM. In DM patients with nephropathy the presence of proteinuria that occurs in 5% to 20% of T2DM patients increases the rate of mortality and morbidity in these patients (3). The prevalence of micro- and macro-albuminuria among Iranian diabetic patients is 16.7%-35.2% (7).

The AT1R gene locates on chromosome 3q21-q25 and consists of 5 exons, 4 of which are untranslated and alternatively spliced. The AT1R A1166C polymorphism (rs5186) in the 3′-untranslated region of AT1R gene may be involved in posttranscriptional modification of AT1R mRNA (8). Some studies have investigated the role of AT1R A1166C polymorphism in susceptibility to DM and DN but with inconsistent results (3,9-12). The present study has investigated the influence of AT1R A1166C polymorphism on the risk of T2DM and its microvascular complication of DN in a population from Western Iran.

## 2. Objectives

The aim of present study was to determine the association between AT1R A1166C variants with the risk of T2DM and DN. Also, we investigated the influence of levels of some biochemical parameters in susceptibility to T2DM and DN in the presence of AT1R variants.

## 3. Patients and Methods

### 3.1. Patients and controls

In a case-control study 135 patients with T2DM and 98 healthy individuals was investigated. Diabetic patients were selected according to the urine albumin to creatinine ratio (ACR) and classified in 3 groups of micro-, macro-, and normo-albuminuria. Patients with severe uncontrolled hypertension (>160/100 mm Hg) were excluded from the study. The mean age of diabetic patients was 55.2 ± 9.3 years that consisted of 46 patients with micro-albuminuria, 48 with macro-albuminuria and 41 with normo-albumiuria. The mean age of healthy subjects was 39.1 ± 14.1 years. The controls were individuals without history of diabetes according to fasting blood sugar that were selected from medical staffs and blood donors. Normo-albuminuric patients were sex matched (12 males and 29 females) with healthy individuals (20 males and 78 females) and with nephropathic patients which consisted of 42 males and 52 females (*P* = 0.25, and *P* = 0.093, respectively). However, nephropathic patients were not sex matched with healthy individuals (*P* < 0.001). The patients had been admitted to the Taleghani Diabetes Research Center of Kermanshah University of Medical Sciences and all of them were from Kermanshah province of Iran with Kurdish ethnic background. T2DM was diagnosed according to World Health Organization (WHO) criteria (13).

The criteria for defining micro-albuminuria and macro-albuminuria were ACR of 30-299 mg/g and ≥300 mg/g, respectively in a random spot collection of urine in three specimens collected within a 3–6 months period. To confirm the presence of micro- or macro-albuminuria in samples with albumin/creatinine ratio (ACR) higher than 30 mg/g, the ACR was measured in 24 hours urine collection. Diabetic patients with ACR <30 mg/g made up the normo-albuminuric patients (14,15).

Detailed medical history of each patient was provided. Informed written consent was obtained from each individual before participation.

### 3.2. Genotype analysis

DNA was extracted from the leukocyte fraction of the EDTA-treated whole blood using the phenol-chloroform method (16).

The AT1R A1166C polymorphism was detected using polymerase chain reaction-restriction fragment length polymorphism (PCR-RFLP). The PCR was conducted using the forward primer of 5′-AAAAGCCAAATCCCACTCAA-3', and the reverse primer of 5′-CAGGACAAAAGCAGGCTAGG-3′. The PCR products were digested with 1.5 U of Dde I restriction enzyme. The presence of AT1R 1166 A allele produced 58- and 374-bp fragments while the AT1R 1166 C allele resulted in 58-, 143-, and 231-bp fragments (17).

### 3.3. Ethical issues

1) The research followed the tenets of the Declaration of Helsinki; 2) informed consent was obtained; and 3) the research was approved by ethical committee of Kermanshah University of Medical Science.

### 3.4. Statistical analysis

The allelic frequencies were calculated by the chromosome counting method. The significance of differences in genotype and allele frequencies of AT1R A1166C between patients and controls were calculated using χ^2^ test. Odds ratios (OR) were calculated as estimates of relative risk for disease and 95% confidence intervals were obtained by SPSS logistic regression software. A 2-tailed *t* test was used to compare quantitative data. Statistical significance was assumed at *P *< 0.05 level. The SPSS version 16 was used for the statistical analysis.

## 4. Results

### 4.1. Study population

The demographic and biochemical characteristics of patients are demonstrated in Table 1. Duration of diabetes and systolic blood pressure were significantly higher (11.9 ± 6.7 years, and 141.4± 18.8 mm Hg, respectively) in macro-albuminuric patients compared to those in normo-albuminuric patients (7.7± 5.7 years, and 132.9 ± 18.7 mm Hg, respectively). Also, in macro-albuminuric patients the concentration of serum creatinine (2.33± 2.55 mg/dl, *P *< 0.001), 24 hours urine albumin excretion (631.3± 507.9 mg/24 h) and ACR (758.2± 686.3 mg/g) were significantly higher than those in normo-albuminuric patients (0.944± 0.18 mg/dl, 23.8± 6.3 mg/24 h, and 23± 6.9 mg/g, respectively). However, in macro-albuminuric patients the level of cholesterol was significantly lower (164± 35.3 mg/dl) than that in normo-albuminuric patients (181.5 ± 27.9 mg/dl) (Table 1).

**Table 1:** Characteristics of diabetic patients with and without nephropathy

**Variables**	**Normo-albuminric patients (n = 41)**	**Micro-albuminuric patients (n = 46)**	**Macro-albuminuric patients (n = 48)**
Age (y)	53±10	54.2±8.8, *P* = 0.55	58.3±8.5, *P* = 0.008
BMI (kg/m^2^)	27.5±4.5	28.3±4.5, *P* = 0.45	26.5±4.4, *P* = 0.26
HbA1c (%)	8.2 ±1.3	8.2 ±1.4, *P* = 0.96	7.7 ±1.5, *P* = 0.16
Diabetes duration (y)	7.7±5.7	7.9±4.8, *P* = 0.85	11.9±6.7, *P* = 0.002
Systolic blood pressure (mm Hg)	132.9±18.7	129±22, *P* = 0.37	141.4±18.8, *P* = 0.037
Diastolic blood pressure (mm Hg)	82.9±10.6	81.1±10.5, *P* = 0.41	85.9±9.2, *P* = 0.15
Creatinine (mg/dl)	0.944±0.18	0.965±0.17, *P* = 0.99	2.33±2.55, *P* < 0.001
24 h urine creatinine excretion (g/24 h)	1.08±0.21	0.98±0.28, *P* = 0.14	0.88±0.22, *P* = 0.003
24 h urine albumin excretion (mg/24 h)	23.8±6.3	107±46.9, *P* = 0.39	631.3±507.9, *P* < 0.001
Albumin/creatinine ratio (mg/g)	23±6.9	107.7±46, *P* = 0.55	758.2±686.3, *P* < 0.001
Triglycerides (mg/dl)	156.7±58	155.4±81.5, *P* = 0.93	157.7±62.7, *P* = 0.94
Cholesterol (mg/dl)	181.5±27.9	176.7±39.7, *P* = 0.52	164±35.3, *P* = 0.012
LDL-C (mg/dl)	99±26	98.2±32.6, *P* = 0.89	102.3±29.7, *P* = 0.58
HDL-C (mg/dl)	44.4±11.8	44.1±14.3, *P* = 0.9	40.5±16.9, *P* = 0.21

All parameters have been compared between micro-albuminuric or macro-albuminuric patients with normo-albuminuric patients.

### 4.2. Distribution of AT1R A1166C variants

Distribution of AT1R A1166C genotypes in all diabetic patients and controls is depicted in Table 2. There was no significant difference in the frequency of AT1R genotypes between patients and the controls (Table 2). Comparing AT1R genotypes in subgroups of diabetic patients demonstrated the frequencies of 19.6%, 29.2%, and 31.7% for AC+CC genotype in micro-, macro- and normo-albuminuric patients, respectively compared to the value of 26.5% in healthy individuals for the same genotype (*P *> 0.05) as demonstrated in Table 3.

**Table 2:** Comparison of the frequency of AT1R genotypes between diabetic patients and the healthy controls

	**Diabetic patients (n = 135)**	**Healthy subjects (n = 98)**
AT1R genotyps		
AA	99 (73.3%)	72 (73.5%)
AC	34 (25.2%)	22 (22.4%)
CC	2 (1.5%)	4 (4.1%)
	(χ^2^* *= 1.66, df* *= 2, *P *= 0.43)	
AC+CC	36 (26.7%)	26 (26.5%)
	(χ^2^* *= 0.001, df* *= 1, *P *= 0.98)	

**Table 3:** The distribution of AT1R A1166C genotypes and alleles in diabetic patients with and without nephropathy

	**T2DM with micro-albuminuria (n=46)**	**T2DM with macro-albuminuria (n=48)**	**T2DM with normo-albuminuria (n=41)**	**Healthy individuals** **(n=98)**
AT1R genotypes				
AA	37 (80.4%)	34 (70.8%)	28 (68.3%)	72 (73.5%)
AC	9 (19.6%)	12 (25%)	13 (31.7%)	22 (22.4%)
CC	0 (0%)	2 (4.2%)	0 (0%)	4 (4.1%)
	*(χ^2^=1.69, df=1, *P *= 0.19) **(χ^2^=2.19, df=2, *P *= 0.33)	*(χ^2^=2.08, df=2, *P *= 0.35) **(χ^2^=0.12, df=2, *P *= 0.94)	**(χ^2^=2.76, df=2, *P *= 0.25)	
AC+CC	9 (19.6%)	14 (29.2%)	13 (31.7%)	26 (26.5%)
OR (95%CI, p)	*0.52 (0.19-1.39, *P *= 0.19) **0.67 (0.28-1.58, *P *= 0.36)	*0.89 (0.35-2.19, *P *= 0.79 **1.14 (0.52-2.45, *P *= 0.73)	**1.29 (0.58-2.85, *P *= 0.53)	
AT1R alleles				
A	83 (90%)	80 (83.5%)	69 (84.1%)	166 (84.7%)
C	9 (10%)	16 (16.5%)	13 (15.9%)	30 (15.3%)
OR (95%CI, p)	**(χ^2^=1.55, df=1, *P *= 0.21) 0.78 (0.52-1.15, *P *= 0.21)	**(χ^2^=0.05, df=1, *P *= 0.82) 1.03 (0.82-1.28, *P *= 0.82)	(χ^2^=0.01, df=1, *P *= 0.9) 0.99 (0.78-1.25, *P *= 0.9)	

*Compared to normo-albuminuric patients. ** Compared to healthy individuals.

### 4.3. AT1R A1166C variants and biochemical parameters

Macro-albuminuric patients with hemoglobin A1c (HbA1c )> 7.5% had a higher frequency of AT1R AC+CC genotype (33.3%) compared to micro-albuminuric patients (12%, *P* = 0.08) and in the presence of uncontrolled hyperglycemia the risk of macro-albuminuria increased 3.66-fold (95% CI: 0.81-16.58, *P* = 0.092) in carriers of AC+CC genotype. Comparison of lipid profile and blood pressure between various genotypes of AT1R A1166C is depicted in Table 4 . As demonstrated in  Table 4 , systolic blood pressure was significantly higher in carriers of CC (170 ± 14.1 mm Hg) genotype compared to AC (132.4 ± 21.6 mm Hg) and AA (134.7 ± 19.6 mm Hg) genotypes in all diabetic patients (*P* = 0.04, and *P* = 0.03, respectively). In DN patients the systolic blood pressure tended to be higher in carriers of CC (170 ± 14.1 mm Hg, *P* = 0.053) than AA genotype (134.6 ± 20.2 mm Hg). Also, serum creatinine level in DN patients was significantly higher in carriers of CC genotype (5.35 ± 5.59 mg/dl) compared to AC (1.59 ± 1.6 mg/dl, *P* = 0.018) and AA (1.58 ± 1.87 mg/dl, *P* = 0.01) genotypes ( Table 4 ). However, due to limited number of patients with CC genotype the obtained results should be considered with caution. In DN patients the presence of mutant allele of 1166 C was associated with lower level of serum HDL-C concentration (36.9 ± 9.8 mg/dl) compared with the value of 44 ± 16.9 mg/dl detected in the presence of wild allele of 1166 A (*P* = 0.058).

**Table 4:** The distribution of AT1R A1166C genotypes and alleles in diabetic patients with and without nephropathy

Parameters	T2DM with nephropathy (n = 93)	All diabetic patients (n = 135)
Systolic blood pressure (mm Hg)		
AA	134.6±20.2	134.7±19.6
AC	134.8±23.4	**132.4±21.6, P = 0.03
CC	*170±14.1, P = 0.053	*170±14.1, P = 0.04
Diastolic blood pressure (mm Hg)		
AA	82.9±9.6	83±10.2
AC	85.5±11.2	83.7±10.1
CC	95±7.1	95±7.1
Triglycerides (mg/dl)		
AA	161.3±78.3	161.7±72.8
AC	14.4±49.2	141.8±50.4
CC	157±70.7	157±70.7
Total cholesterol (mg/dl)		
AA	171.8±38.7	175.1±36.3
AC	163.8±36.1	168.8±32.9
CC	185±46.7	185±46.7
HDL-C (mg/dl)		
AA	44.1±16.9	43.9±15.8
AC	37.4±10.1	40.7±10.8
CC	32±5.7	32±5.7
LDL-C (mg/dl)		
AA	101.3±31.2	100.5±29.9
AC	94.6±26.9	96.2±25.4
CC	135±67.8	135±67.8, *P = 0.001
Creatinine (mg/dl)		
AA	1.58±1.87	1.4±1.59
AC	1.59±1.6	1.33±1.29
CC	5.35±5.59, *P = 0.01, ***P = 0.018	5.35±5.59, *P = 0.001, ***P = 0.001

*Compared to AA genotype, ** compared to CC genotype, *** compared to AC genotype.

### 4.4. AT1R A1166C variants and diabetes complications

All diabetic and DN patients with retinopathy had higher frequency of AT1R AC+CC genotype (32% and 29.7%, respectively) compared to those without retinopathy and with the same genotype (23.8%, *P* = 0.3 and 21.8%, *P* = 0.39, respectively). Also, in DM and DN patients with neuropathy a higher frequency of AC+CC genotype (31.2% and 30.2%, respectively) was detected compared to those patients without neuropathy (20%, *P* = 0.14 and 17.5%, *P* = 0.16, respectively).

## 5. Discussion

There are ethnic differences in the development of DN leading to a higher development of DN and ESRD among T2DM patients from South Asia compared to those from Europe (18).

Ang II, results from enzymatic reaction of angiotensin converting enzyme (ACE) on angiotensin I, binds to AT1R and through this receptor performs its actions (19).

The present study indicates a higher but not a significantly different frequency of AT1R AC+CC genotype in normo-albuminuric and macro-albuminuric patients compared to controls. In micro-albuminuric patients, the frequency of AT1R AC+CC was lower than the normo-albuminuric ones and the controls (*P* > 0.05). There are controversial reports, even within a country, related to the role of AT1R A1166C polymorphism in the risk of developing T2DM and DN. In a large study of Caucasian the AT1R A1166C was found to be a risk factor for progression of renal disease to ESRD and a predictor for early ESRD (20). Further, in Caucasian with T2DM, the AT1R 1166CC genotype increased the risk of macro-albuminuria only in men (21). In large samples of Japanese T2DM the AT1R A1166C affected the progression of renal failure, especially in females (22). Two out of three studies from India which examined the role of this polymorphism on the risk of T2DM and its complications reported an association between AT1R C allele with T2DM and the higher risk of DN (9,10). Among Tunisian diabetic patients, a significant association has been demonstrated between CC genotype or C allele of AT1R A1166C polymorphism and increased risk of T2DM (3). Two recent meta-analyses suggest that the AT1R A1166C polymorphism may contribute to DN development, particularly in T2DM patients with a significant association for CC versus AA model (12,23). The presence of these associations might be due to higher expression of AT1R in the presence of 1166 C allele and higher affinity of resulted receptor variant for Ang II (3). The enhanced Ang II action resulted in increase kidney susceptibility to the effects of hyperglycemia through abnormalities of systemic or renal hemodynamics, or by altering the function of renal cells (23). However, the lack of association between AT1R A1166C with the risk of T2DM or DN among Indians (3), Taiwanese (24), and Mexican-American families (25) has been reported. In contrast, in a report from Caucasian the wild genotype of AT1R 1166 AA increased the risk of overt DN in T1DM by 3-fold and the risk more increased while the smoking was present (26). The inconsistent findings suggest that a more complex model consisting of still poorly understood combinations of several RAAS gene variants may affect disease susceptibility (10). Also, ethnicity, definition of DN and its different stages, difference in sample size, gene-gene and gene-environment interactions could affect the results of different studies (21).

Ang II is a potent vasoconstrictor and it stimulates salt and water retention leading to systemic hypertension which is a major risk factor for DN. The AT1R mediates vasoconstriction and the proliferative action of Ang II (27). Several studies have reported the association of AT1R gene polymorphism and the development of hypertension and this mutation predicts the response to Ang II blockers, thus strengthening the detrimental effect of A1166C mutation (10). In the present study diabetic patients with AT1R CC genotype had significantly higher systolic blood pressure compared to AC and AA genotypes carriers that needs to be confirmed in studies with larger samples. Also, affecting this polymorphism on the higher serum level of creatinine in the presence of C allele than A allele should be confirmed in larger samples.

In the study of Doria et al (28), an association between AT1R A1166C polymorphism and the susceptibility to DN in the presence of hyperglycemia in T1DM patients was demonstrated. They suggested that there might be a synergistic effect between hyperglycemia and the C allele of AT1R on the risk of kidney damage through similar mechanisms of signal transduction pathways. In the present study we observed a trend toward an increased risk of developing macro-albuminuria in the presence of AC+CC genotype in those patients with HbA1c>7.5%.

The present study detected a nonsignificant higher frequency of AC+CC genotype in those diabetic patients with neuropathy than those without neuropathy. The level of Ang II that enhances in the presence of hyperglycemia stimulates NAD (P) - oxidase which increases oxidative stress and vascular damage leading to diabetic neuropathy (29). Also, disturbance in the metabolism and vasculature of nerve tissue in the presence of excessive uptake of glucose might lead to neuropathy (30). The role of AT1R A1166C polymorphism on the risk of neuropathy needs to be elucidated.

A nonsignificantly higher frequency of AC+CC genotype was found in our studied patients with retinopathy compared to those without retinopathy. Damage of retinal vasculature by hyperglycemia, hypertension and hyperlipidemia are involved in the pathogenesis of diabetic retinopathy. Both ACE and Ang II increases the level of vascular endothelial growth factors leading to abnormal retinal angiogenesis and increased risk of retinopathy and its progression. Both systemic and local RAAS are implicated in the pathogenesis of retinopathy and all components of the RAAS are expressed in retina with highly elevation of renin, ACE, Ang II and AT1R in patients with diabetic retinopathy (31). In one available study among Caucasians with T1DM the AT1R A1166C was not associated with the risk of proliferative retinopathy (32). More studies necessary to establish a possible role for AT1R A1166C in the pathogenesis of diabetic retinopathy.

## 6. Conclusions

In summary, our study indicated a nonsignificantly increased and decreased frequency of AT1R AC+CC genotype in normo-albuminuric and macro-albuminuric patients, respectively and a nonsignificantly decreased frequency of this genotype in micro-albuminuric patients compared to controls. We observed that AT1R AC+CC genotype tended to increase the risk of macro-albuminuria in the presence of HbA1c >7.5%. Also, a higher frequency of AC+CC genotype was observed in diabetic patients with neuropathy or retinopathy compared with those without neuropathy and retinopathy. It seems in carriers of 1166 C allele systolic blood pressure and serum creatinine level to be higher compared to the 1166 A allele carriers. We observed a trend toward decreased HDL-C level in the presence of AT1R C compared with AT1R A allele in DN patients.

## 7. Limitations of the study

The limitation of the present study is the small sample sizes of the study groups, mainly the control group.

## Authors’ contribution

MM contributed to drafting and revising the manuscript. ZR contributed to the conception and design of the work, drafting and revising the manuscript. SA and ZR performed the experiments. MV and HN critically revised the manuscript.

## Conflicts of interest

The authors declared no competing interests.

## Funding/Support

This research was supported by Kermanshah University of Medical Sciences (grant No. 91246).

## References

[1] Atkins RC, Zimmet P (2010). Diabetic kidney disease: act now or pay later. IJKD.

[2] Motavallian A, Andalib S, Vaseghi G, Mirmohammad-Sadeghi H, Amini M (2013). Association between PRO12ALA polymorphism of the PPAR-γ2 gene and type 2 diabetes mellitus in Iranian patients. Indian J Hum Genet.

[3] Mehri S, Koubaa N, Hammami S, Mahjoub S, Chaaba R, Nakbi A (2010). Genotypic interactions of renin–angiotensin system genes with diabetes type 2 in a Tunisian population. Life Sci.

[4] Ruggenenti P, Bettinaglio P, Pinares F, Remuzzi G (2008). Angiotensin converting enzyme insertion/deletion polymorphism and renoprotection in diabetic and nondiabetic nephropathies. Clin J Am Soc Nephrol.

[5] Abd El-Aziz TA, Hussein YM, Mohamed RH, Shalaby SM (2012). Renin–angiotensin system genes polymorphism in Egyptians with premature coronary artery disease. Gene.

[6] Chawla T, Sharma D, Singh A (2010). Role of the renin angiotensin system in diabetic nephropathy. World J diabetes.

[7] Zakerkish M, Shahbazian HB, Shahbazian H, Latifi SM, Aleali AM (2013). Albuminuria and its correlates in type 2 diabetic patients. IJKD.

[8] Alfakih K, Lawrance RA, Maqbool A, Walters K, Ball SG, Balmforth AJ (2005). The clinical significance of a common, functional, X-linked angiotensin II type 2-receptor gene polymorphism (1332 G/A) in a cohort of 509 families with premature coronary artery disease. Eur Heart J.

[9] Ahluwalia TS, Ahuja M, Rai TS, Kohli HS, Bhansali A, Sud K (2009). ACE variants interact with the RAS pathway to confer risk and protection against type 2 diabetic nephropathy. DNA Cell Biol.

[10] Shah VN, Cheema BS, Sharma R, Khullar M, Kohli HS, Ahluwalia TS (2013). ACACβ gene (rs2268388) and AGTR1 gene (rs5186) polymorphism and the risk of nephropathy in Asian Indian patients with type 2 diabetes. Mol Cell Biochem.

[11] Prasad P, Tiwari AK, Kumar KMP, Ammini AC, Gupta A, Gupta R (2006). Chronic renal insufficiency among Asian Indians with type 2 diabetes: I Role of RAAS gene polymorphisms. BMC Med Gen.

[12] Ding W, Wang F, Fang Q, Zhang M, Chen J, Gu Y (2012). Association between two genetic polymorphisms of the renin-angiotensin-aldosterone system and diabetic nephropathy: a meta-analysis. Mol Biol Rep.

[13] WHO Study Group Report of a WHO Consultation. Part 1. Diagnosis and Classification of Diabetes Mellitus. Geneva: World Health Organization; 1999.

[14] Rahimi Z, Felehgari V, Rahimi M, Mozafari H, Yari K, Vaisi-Raygani A (2011). The frequency of factor V Leiden mutation, ACE gene polymorphism, serum ACE activity and response to ACE inhibitor and angiotensin II receptor antagonist drugs in Iranians type II diabetic patients with microalbuminuria. Mol Biol Rep.

[15] Jafari Y, Rahimi Z, Vaisi-Raygani A, Rezaei M (2011). Interaction of eNOS polymorphism with MTHFR variants increase the risk of diabetic nephropathy and its progression in type 2 diabetes mellitus patients. Mol Cell Biochem.

[16] Rahimi Z, Akramipour R, Nagel RL, Ahmadi AS, Merat A, Bahrehmand F (2006). The beta-globin gene haplotypes associated with Hb D-Los Angeles [beta121(GH4) Glu --> Gln] in Western Iran. Hemoglobin.

[17] Rahimi Z, Rahimi Z, Mozafari H (2013). Parsian A Preeclampsia and angiotensin converting enzyme (ACE) I/D and angiotensin II type-1 receptor (AT1R) A1166C polymorphisms: association with ACE I/D polymorphism. J Renin Angiotensin Aldosterone Syst.

[18] Rahimi Z, Rahimi Z, Shahvaisi-Zadeh F, Sadeghei S, Vessal M, Yavari N (2013). eNOS 4a/b polymorphism and its interaction with eNOS G894T variants in type 2 diabetes mellitus: Modifying the risk of diabetic nephropathy. Dis Markers.

[19] Rahimi Z (2012). ACE insertion/deletion (I/D) polymorphism and diabetic nephropathy. J Nephropathol.

[20] Buraczynska M, Ksiazek P, Drop W, Zaluska W, Spasiewicz D, Ksiazek A (2006). Genetic polymorphisms of the renin angiotensin system in end-stage renal disease. Nephrol Dial Transplant.

[21] Fradin S, Goulet-Salmon B, Chantepie M, Grandhomme F, Morello R, Jauzac P (2002). Relationship between polymorphisms in the renin-angiotensin system and nephropathy in type 2 diabetic patients. Diabetes Metab.

[22] Tomino Y, Makita Y, Shike T, Gohda T, Haneda M, Kikkawa R (1999). Relationship between polymorphism in the angiotensinogen, angiotensin converting enzyme or angiotesin converting enzyme or angiotensin II receptor and renal progression in Japanese NIDDM patients. Nephron.

[23] Wang F, Fang Q, Yu N, Zhao D, Zhang Y, Wang J (2012). Association between genetic polymorphism of the angiotensin-converting enzyme and diabetic nephropathy: a meta-analysis comprising 26580 subjects. J Renin Angiotensin Aldosterone Syst.

[24] Chang HR, Cheng CH, Shu KH, Chen CH, Lian JD, Wu MY (2003). Study of the polymorphism of angiotensinogen, angiotensin-converting enzyme and angiotensin receptor in type II diabetes with end-stage renal disease in Taiwan. J Chin Med Assoc.

[25] Thameem F, Puppala S, Arar N, Blangero J, Stern MP, Duggirala R (2008). Genetic polymorphisms in the renin angiotensin system (RAS) genes and their association analysis with type 2 diabetes and related traits in Mexican Americans. Diabetes Res Clin Practice.

[26] Mollsten A, Kockum I, Svensson M, Rudberg S, Ugarph-Morawski A, Brismar K (2008). The effect of polymorphisms in the renin–angiotensin–aldosterone system on diabetic nephropathy risk. J Diabetes Complications.

[27] Rahimi Z, Mansouri-Zaveleh O, Rahimi Z, Abbasi A (2013). AT2R -1332 G:A polymorphism and diabetic nephropathy in type 2 diabetes mellitus patients. J Renal Inj Prev.

[28] Doria A, Onuma T, Warram JH, Krolewski AS (1997). Synergistic effect of angiotensin II type I receptor genotype and poor glycaemic control on risk of nephropathy in IDDM. Diabetologia.

[29] Seshiah PN, Weber DS, Rocic P, Valppu L, Taniyama Y, Griendling KK (2002). Angiotensin II stimulation of NAD (P) H oxidase activity. Circ Res.

[30] Mansoor Q, Javaid A, Bilal N, Ismail M (2012). Angiotensin-converting enzyme (ACE) gene II genotype protects against the development of diabetic peripheral neuropathy in type 2 diabetes mellitus. J Diabetes.

[31] Shah CA (2008). Diabetic retinopathy: a comprehensive review. Indian J Med Sci.

[32] Tarnow L, Cambien F, Rossing P, Nielsen FS, Hansen BV, Ricard S (1996). Angiotensin-II type 1 receptor gene polymorphism and diabetic microangiopathy. Nephrol Dial Transplant.

